# Why expanding public health insurance coverage is not enough to provide effective ambulatory care: policy lessons from Mexico, 2000–2022

**DOI:** 10.1057/s41271-025-00564-y

**Published:** 2025-05-29

**Authors:** Adolfo Martínez-Valle

**Affiliations:** Centro de Investigación en Políticas, Población y Salud, Facultad de Medicina, Universidad Nacional Autónoma de México, Ciudad Universitaria, Edificio CIPPS Piso 2, Centro Cultural, Coyoacán, 04510 Mexico City, Mexico

**Keywords:** Private use, Outpatient health care, Public health insurance, Policy lessons, Mexico

## Abstract

Despite expanding public insurance coverage and investment in public healthcare supply, the Mexican population not covered by social security has increasingly used private-sector outpatient health services over the past two decades. This is a public health policy problem because Mexico is committed to a constitutional right to health protection, which means unmet ambulatory needs must be fulfilled. This brief aims to measure the magnitude of unmet ambulatory health care needs, analyze factors that led to their growth, and formulate policy options to address them. Private services’ share of total ambulatory care grew from 38 percent in 2006 to 66 percent in 2022, despite two national policy efforts to increase public coverage to nearly 50 million people. Neither policy provided adequate ambulatory coverage for its targeted population, leading to care seeking through private outpatient providers. We recommend strengthening public ambulatory care by increasing financial resources for public primary care and implementing more effective allocation to improve timeliness and quality of care.

## Key messages

- Increasing public health insurance coverage leads to high private health care use if untimely and low quality ambulatory public health care is provided.
- Effective public health spending is necessary to provide timely and adequate ambulatory public health care by improving the capacity of public ambulatory health care units.
- Private ambulatory health care capacity should be used with effective regulatory frameworks when sufficient allocation of public health resources is not feasible.

## Context

Over nearly the past two decades, the Mexican population without social security has increasingly used ambulatory private-sector health services despite two national public policy efforts, Seguro Popular (SP) and the National Health Institute for Welfare (Insabi), implemented to guarantee access to public ambulatory care to almost fifty million people. This remains a policy problem because their constitutional right to public ambulatory healthcare coverage has yet to be exercised. We aim to measure the magnitude of this problem, analyze the factors that led to its growth, and formulate policy options to address it.

SP was an evidence-based public national health insurance model implemented between 2003 and 2019 to provide financial protection and explicit health care coverage. Insabi was a national health system model created in 2019 to replace SP based on political and ideological grounds. It was argued that SP failed to provide free and universal public health care coverage because it fostered corruption and “neoliberal” practices, including privatization [1]. Insabi assumed that eliminating these practices would be sufficient to provide the necessary funding for providing universal health coverage. SP financed an essential health care package, including tertiary care and drug coverage based on the number of people affiliated with its insurance scheme [2, 3]. However, Insabi provided in practice less coverage, excluding tertiary care, without clearly defining how it was financed. Decentralized state-level services provided health care funded by SP, while Insabi centralized financing and health care provision to reduce corruption at the state level [2, 3].

**Fig. 1:**
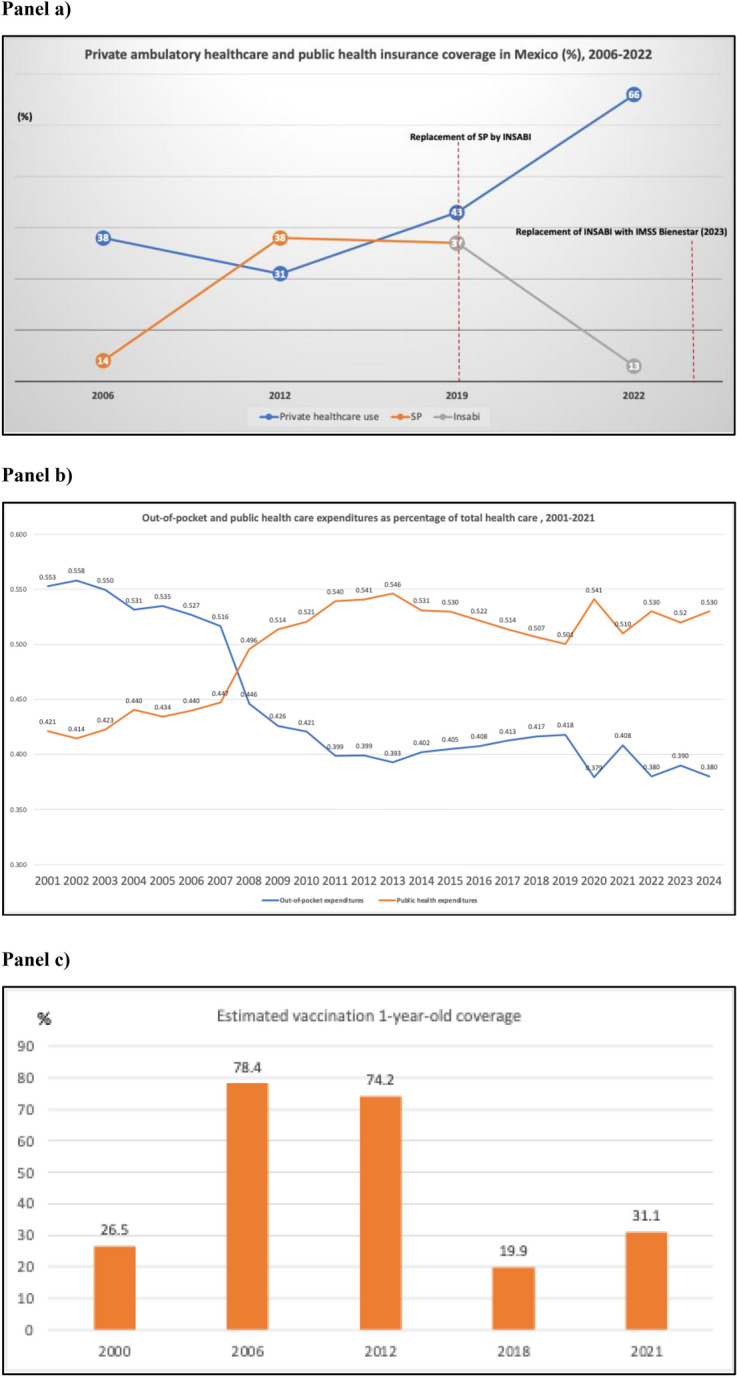
The trends in key characteristics: Panel (**a**) the use of private ambulatory care in 2006–2022 [5, 6, 7, 8, 9]; Panel (**b**) out-of-pocket and catastrophic health expenditures in 2001–2024 [10]; Panel (**c**) the vaccination coverage in 2000–2021 [11]

Fig. 1, panel (a) shows the trends in the use of private ambulatory care by the population without social security compared to its public insurance coverage between 2006 and 2022. During Seguro Popular (SP), the Mexican government adopted a public health insurance scheme to cover this unprotected population, and private ambulatory use decreased from 38% in 2006, after 4 years of implementation, to 31% in 2012. Then, it grew to 43% in 2018, a year before Insabi replaced SP. In the same period, SP nearly doubled its public insurance coverage from 14% of the total population in 2006 to 37% in 2018. With Insabi, private ambulatory health care grew to 66% after 3 years of implementation, and coverage dropped to 13% of the total population in 2022. After failing to meet its objectives, Insabi was replaced with IMSS Bienestar, a new national agency affiliated with Mexico’s leading social security institution, the Mexican Institute of Social Security (IMSS) [4].

## Takeaways

Expanding public insurance coverage proved insufficient to meet all inpatient healthcare needs throughout SP’s years of implementation. As its coverage increased, public expenditure as a percentage of total health expenditure grew steadily from 43 in 2003 to 55 in 2013, as Fig. 1, panel (b) shows [6]. It managed to reduce out-of-pocket and catastrophic health expenditures [12–17], improved access to preventive care [18–22], and increased utilization and treatment [23–28]. These effects were associated with lower use of private care between 2006 and 2012 by the SP-insured population. However, since 2012, SP coverage diminished slightly, and private ambulatory healthcare use increased among SPS users until its substitution by Insabi in 2019. This policy decision was mainly based on ideological and political grounds. It was argued that SP was neither “Seguro” because it did not financially protect people from illness nor “Popular” because people did not affiliate with its insurance scheme, despite the evidence of its positive effects [2, 3, 14].

Insabi eliminated the insurance features of SP, expecting to ensure access to quality and timely ambulatory care in public health facilities by removing corruption and inefficient practices assuming additional resources were not necessary [1–3, 14]. However, Insabi failed to do so as private ambulatory healthcare grew to its highest percentage, and public coverage diminished to its lowest in 2022 (Panel a). Furthermore, public health expenditures fell from 55% of total health expenditures in 2020 to 51 in 2021, a percentage not registered since 2009, as Panel (b) shows. However, in 2024, it rose slightly but was still far behind its highest 55% nearly a decade ago.

Fig. 1, panel (c) shows the vaccination coverage in the 1-year-old population during the implementation of SP and Insabi. With SP in place, vaccination coverage of BCG, Hepatitis B, Pentavalent, and SRP reached its highest level between 2006 and 2012 and dropped to a low level of 31% in 2021 with Insabi. This is related to a lack of effective primary care, lower demand, insufficient vaccines, lower expenditures, ineffective stewardship [29, 30], and lack of health insurance [31]. The lowest level, 20%, is attributable to the worldwide scarcity of vaccines [32, 33].

According to the available literature from Mexico [11, 34–37] and other middle-income countries [38–40], this population, which should have access to public ambulatory services, sought care in the private sector for three reasons:

- These insured public users seek more accessible private health care due to their extended hours of operation and geographical location [11, 34].
- They also seek private, more timely health care for acute problems such as stomach pain or everyday respiratory infections [11].
- Users perceive private care as higher quality [11, 34], including drug availability, as shown in other countries [38–40].

## Conclusions

The trends of private ambulatory services used by the population without social security in Mexico over the past 20 years suggest that SP public insurance coverage provided limited access to ambulatory public health care during its nearly two decades of implementation until its replacement by Insabi in 2019. However, the higher use of private ambulatory services with Insabi indicated even less access to public ambulatory care. This failed policy led to a new strategy: replacing Insabi with IMSS Bienestar in 2023.

The scope of this policy brief is limited. It analyzed trends only and corroborated results using available literature without performing a complete quantitative multi-factor analysis to more precisely identify factors attributable to the growth of ambulatory care use. Further research is needed to identify specific mechanisms for implementing more effective public policies to regulate and capitalize on private ambulatory units’ capacity to provide acute care. Also, IMSS Bienestar should be rigorously monitored and evaluated to assess its effectiveness.

Despite these limitations, the findings of this policy brief helped identify windows of opportunity to improve the provision of public ambulatory services through IMSS Bienestar in Mexico and other public strengthening strategies in different policy settings like Mexico. These suggest a systemic approach, which entails jointly addressing the Mexican public health system’s financing, provision, and stewardship functions. Financing would require additional public investment and more effective spending to improve the capacity of public outpatient establishments to provide needed care. The provision of timely and high-quality ambulatory public services would be expected of this adequate allocation of resources. This, in turn, would require a vital stewardship function from the Ministry of Health through an effective  monitoring and evaluation role.

## Data Availability

Not applicable.
